# ssHiCstuff: a package for the design and analysis of ssDNA-specific Hi-C experiments

**DOI:** 10.1093/bioinformatics/btag417

**Published:** 2026-06-22

**Authors:** Nicolas Mendiboure, Laurent Modolo, Stéphane Janczarski, Aurèle Piazza

**Affiliations:** Laboratoire de Biologie et Modélisation de la Cellule, ENS de Lyon, CNRS UMR5239, Université de Lyon, Université Claude Bernard, 46 Allée d’Italie, Lyon 69007, France; Laboratoire de Biologie et Modélisation de la Cellule, ENS de Lyon, CNRS UMR5239, Université de Lyon, Université Claude Bernard, 46 Allée d’Italie, Lyon 69007, France; Laboratoire de Biologie et Modélisation de la Cellule, ENS de Lyon, CNRS UMR5239, Université de Lyon, Université Claude Bernard, 46 Allée d’Italie, Lyon 69007, France; Laboratoire de Biologie et Modélisation de la Cellule, ENS de Lyon, CNRS UMR5239, Université de Lyon, Université Claude Bernard, 46 Allée d’Italie, Lyon 69007, France

## Abstract

**Motivation:**

Single-strand DNA-specific Hi-C (ssHi-C) is a recently developed technique enabling the capture of chromatin interactions involving single-stranded DNA (ssDNA), an intermediate of various DNA metabolic processes. ssHi-C entails the restoration of restriction sites in ssDNA regions of interest upon introduction of designer, internally barcoded “annealing oligonucleotides” prior to the restriction digestion step of Hi-C. The design of these “annealing oligonucleotides,” as well as the analysis of the resulting ssHi-C data presents specific challenges, such as (i) differentiating ssDNA from dsDNA-derived contacts, (ii) tracking probe-specific interactions, and (iii) calibrating the amount of ssDNA contacts across biological samples. Dedicated computational tools are therefore needed to facilitate the design of, and extract biological information from, ssHi-C experiments.

**Results:**

We present ssHiCstuff, a Rust- and Python-based package for the design of key reagents for ssHi-C experiments and for the analysis of ssHi-C data. ssHiCstuff provides (i) an automated annealing oligonucleotides design module, (ii) an end-to-end analyses pipeline, and (iii) a graphical user interface. ssHiCstuff simplifies the high-resolution analysis of ssDNA interactions at genome-wide scale. A graphical user interface (GUI) implemented in Python is also available for biologists without coding skills.

**Availability:**

ssHiCstuff is freely available at https://github.com/Piazzalab/ssHiCstuff and https://zenodo.org/records/19677479 (https://doi.org/10.5281/zenodo.19677479) under the GPL 3.0 license. The annealing oligonucleotides design and the visualization modules are additionally freely available on a web browser at https://bioshiny.ens-lyon.fr/public/app/sshicstuff. A test dataset is available at https://zenodo.org/records/20035366 (https://doi.org/10.5281/zenodo.20035366).

## 1 Introduction

High-throughput chromosome conformation capture approaches, and most prominently Hi-C (Lieberman-Aiden *et al.* 2009), are central to the field studying the mechanisms and functions of 3D chromatin organization. Hi-C relies on proximity ligation between double-stranded DNA (dsDNA) ends, making it blind to genomic regions in a single-stranded (ssDNA) state, a hallmark of several universal DNA metabolic processes such as DNA replication and recombination. Indeed, hundred to kilobases-long ssDNA are generated by specialized nucleases for DNA break repair or replication fork restart (Ceccaldi and Cejka 2025). These ssDNA regions can be harnessed as guides by RecA/Rad51-family proteins to perform homology search and identify homologous repair templates (Raina *et al*. 2025). ssDNA also arises during lagging strand synthesis, mitochondrial replication, break-induced replication, as intermediates in various viral and phage replication, as well as during bacterial transformation. Detecting ssDNA-mediated chromatin contacts genome-wide would enable determining the nuclear context of these active processes.

We recently developed ssHi-C, a ssDNA-specific Hi-C variant, to study homologous recombination in *S. cerevisiae* (Dumont *et al*. 2024). ssHi-C restores dsDNA at Hi-C restriction sites using custom “annealing oligonucleotides” prior to digestion and ligation, followed by targeted enrichment using “capture oligonucleotides.” Optionally, annealing oligos can introduce mutations in secondary restriction sites, allowing to enzymatically eliminate dsDNA signals at these sites, in addition to bioinformatically parse reads based on SNPs content.

This method enables to isolate chromatin contacts made by ssDNA sites, even if present in only a subset of cells. Two barriers currently limit the broader adoption of ssHi-C: the lack of automated tools for oligonucleotide design, and the absence of dedicated analysis pipelines to isolate ssDNA contacts from dsDNA contacts.

## 2 Methods

We developed ssHiCstuff, a dedicated Rust- and Python-based package, for end-to-end design and analysis of ssHi-C experiments. ssHiCstuff is built upon the HiCstuff package (Matthey-Doret *et al*. 2020, 2022). Our pipeline is composed of several modules, detailed in the subsections below, going from the design of annealing oligonucleotides to the visualization and quantification of contacts made by individual ssDNA sites. ssHiCstuff offers a reproducible, flexible framework tailored for the analysis of ssDNA-derived chromatin interaction data, enabling new insights into DNA metabolism beyond the reach of conventional Hi-C.

### 2.1 Oligonucleotides design and edition of the reference genome

**Figure 1:**
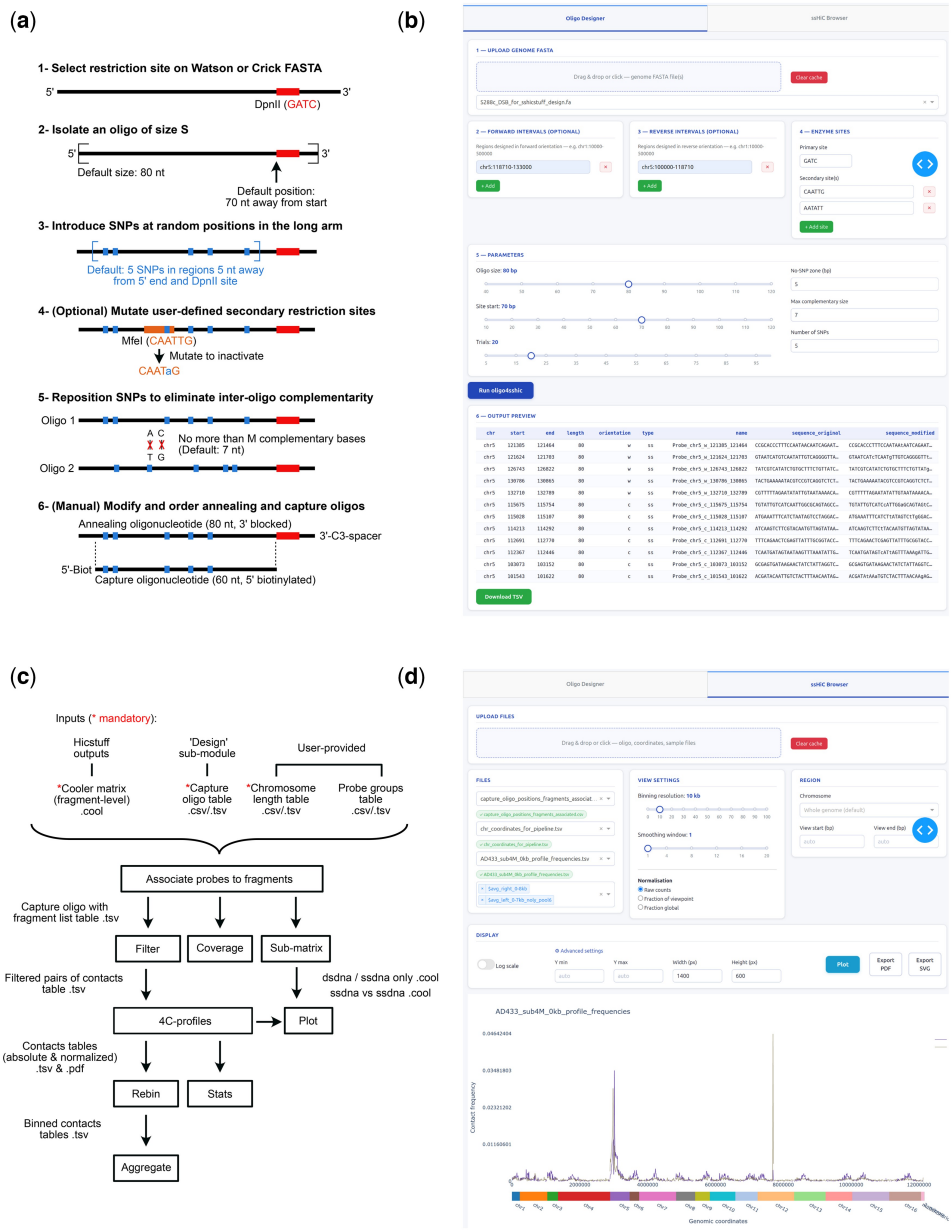
Overview of the ssHiCstuff package. (a) The ssHiCstuff “*design*” module generates oligonucleotides to anneal to a specific strand (Watson or Crick) to restore a restriction site (e.g. DpnII) within user-specified genomic intervals. It iteratively introduces SNPs, mutates secondary restriction sites, and checks for absence of complementarity between the pool of generated oligonucleotides. (b) Overview of the ssHiCstuff “*analysis*” pipeline, from the output of sequencing read alignment and sparse Hi-C matrix generation with HiCstuff. (c) GUI of the “*design*” module. (d) GUI for the interactive visualization of 4C-like genome-wide contacts of individual or grouped ssDNA sites.

ssHi-C entails the restoration of double-stranded DNA at ssDNA regions for the restriction digestion step of the Hi-C protocol (Dumont *et al*. 2024). This is achieved by annealing synthetic oligonucleotides that recreate a primary restriction site (*e.g.* DpnII) while introducing specific sequence modifications (Fig. 1a). These modifications can be used to isolate ssDNA contacts at the alignment step of the pipeline. Optionally, these sequence modifications can mutate secondary restriction sites, making ssDNA-derived contacts resistant to the digestion with the corresponding restriction enzyme (e.g. MfeI or SspI) (Dumont *et al*. 2024).

The “design” module formalizes the empirical “know-how” and automates the generation of the annealing and capture oligonucleotides. Given a genome FASTA file and a series of user-specified genomic intervals, it selects non-overlapping sequences that (i) restore a user-defined primary restriction site (e.g., DpnII), (ii) introduce several single-nucleotide polymorphisms (SNPs), and (iii) maximize genome coverage while minimizing unwanted overlaps or complementarity between oligonucleotides (Fig. 1a). Moreover, upon building the oligonucleotides, the module also edits the reference genome FASTA file in order to distinguish ssDNA from dsDNA contacts at the reads mapping step of the pipeline. The resulting FASTA contains additional *chr_artificial_ssDNA* and *chr_artificial_dsDNA* sequences that consist of the concatenation of the annealing oligonucleotides sequence or of their genomic targets, respectively. The target of these oligonucleotides, present in *chr_artificial_dsDNA*, are also eliminated from their native chromosomal site. This modified reference allows for competitive mapping of paired-end sequencing reads from either dsDNA or ssDNA origins, and thus to parse contacts originated from dsDNA and ssDNA molecules at the sites of interest.

### 2.2 Analysis of ssHi-C data

#### 2.2.1 Inputs

The raw 2 × 150 bp paired-end Illumina reads are aligned and the contacts processed with the standard hicstuff pipeline (with the—cutsite and—filter modes enabled) using the reference genome produced by the “*design*” module of ssHiCstuff. This first step results in a 6-column genome restriction fragment list (fragment_id, chromosome, start, end, size, gc_content) and a contact matrix. ssHiCstuff accepts contact matrices in Cooler format (*.cool*) (Abdennur and Mirny 2020) at fragment resolution as its primary input. Legacy GRAAL sparse matrix files (*.tsv* or .*txt*) produced by hicstuff remain supported and are automatically converted to .cool via the “*graal2cool*” sub-command or transparently at pipeline initialization. ssHiCstuff requires 3 additional inputs to extract and analyze contacts specific to single-stranded DNA regions (Fig. 1b):

- The list of oligonucleotides generated by the “design” module.
- A user-generated 4-columns table containing chromosome metadata ([chr, length, left_arm_length, right_arm_length]).
- Optionally, a 3-columns table with groups of ssDNA sites to sum or average ([name, probes, action]).

#### 2.2.2. Pipeline

ssHiCstuff consists of over a dozen modular sub commands, some *essential (e.g. filtering, profiling), others optional or utility-focused (e.g.* plotting, merging). Each sub command can be run independently, provided the required input files are correctly formatted (*c.f.* README.md). For ease of use and reproducibility, we provide a unified “*pipeline*” command that sequentially executes the main steps of the analysis from oligo-to-fragment association, through filtering, coverage calculation, 4C-like profile generation, and re-binning, statistics, up to the final aggregation of contacts around user-specified features (*e.g.* centromeres and telomeres) (Fig. 1b). Each pipeline step can be called independently with the following sub-modules:

#### 2.2.3. Filter, normalize and extract coverage

ssHiCstuff provides several tools to selectively process contact matrices. The “*ssdnaonly***”** and “*dsdnaonly***”** sub commands allow users to filter the sparse matrix by retaining only contacts involving, respectively, ssDNA-ssDNA or dsDNA-dsDNA (*i.e.* regular Hi-C) contacts, producing new *.cool* files. To address systematic technical biases (GC content, fragment length, mappability), ssHiCstuff implements iterative correction (ICE) via the “*balance*” sub-command. These operations generate new sparse matrices. The “*coverage*” command computes per-fragment contact coverage and outputs it in a standard bedgraph format. Finally, the “*filter*” sub-command retains all contacts involving at least one probe-associated fragment (ssDNA or dsDNA control), producing both (i) a filtered *.cool* file and (ii) a “dense” tab-delimited table with all genomic fragments listed (including zero-containing bins) used as input by the subsequent “*profile*” command.

#### 2.2.4. Visualization

From the filtered contact table, ssHiCstuff can generate 4C-like genomic profiles using the “*profile*” command. These profiles represent contact frequencies between a given ssDNA fragment and the rest of the genome, providing a one-dimensional view of interaction patterns. The “*rebin***”** command allows users to adjust the genomic resolution by changing the bin size, and the “*plot4C*” command visualizes these profiles with optional smoothing or logarithmic scaling.

To explore interactions between probes themselves, the “*probe2probe*” command generates square heatmaps displaying contact frequencies between all pairs of ssDNA fragments.

#### 2.2.5. Aggregate

To facilitate the interpretation of 4C-like profiles at a population scale, ssHiCstuff allows aggregation of probe signals around genomic landmarks using the “*aggregate*” command. By default, the pipeline uses predefined centromere and telomere coordinates for *S. cerevisiae*, but users can provide custom genomic features in BED-like format. The command computes average contact profiles across all probes or user-defined probe groups relative to the specified landmarks, enabling comparisons of interaction patterns across genomic contexts.

#### 2.2.6. Statistics and comparison

A central feature of ssHiCstuff is the ability to quantify, for each probe, how and where it interacts with the genome. The “*stats*” command takes as input the unbinned contact table and the initial contact matrix in Cooler format. It computes, for every probe, the total number of contacts it makes and the proportion these represent over the full Hi-C dataset, providing a normalized measure of coverage. This measure of ssDNA capture efficiency is normalized against that of control dsDNA sites captured alongside ssDNA targets. Such metrics enables direct comparison of ssDNA-specific enrichment across experiments by using the sub command “*compare*,” which performs ratio of normalized capture efficiency between two samples. This function uniquely allows to calibrate ssDNA contacts across samples, providing a readout for differences in ssDNA proficiency for engaging other DNA molecules between two biological conditions.

It then classifies each probe’s contacts based on their genomic origin: whether they occur within the same chromosome (intra-chromosomal) or across different chromosomes (inter-chromosomal), and whether they are within a defined local window (*cis*) or outside of it (*trans*). These contact distributions are further normalized by chromosome length. Together, these statistics enable quick quality control, identification of preferential genomic interaction patterns, and normalization of capture efficiency across samples.

### 2.3 Graphical user interface

We developed a lightweight web-based interface using Dash and Flask (Plotly Technologies Inc 2015), organized into two main modules. The Design tab provides a graphical front-end for oligonucleotide generation, allowing users to visualize and export candidate probes (Fig. 1c). The browser tab enables interactive exploration of 4C-like contact profiles, including zooming, binning, smoothing, and multi-probe and multi-sample comparisons (Fig. 1d). The platform runs as a stand-alone Flask server hosted on our institutional infrastructure, allowing remote access without requiring local installation at https://bioshiny.ens-lyon.fr/public/app/sshicstuff.

## 3 Discussion

The ssHi-C methodology enabled genome-wide mapping of chromatin contacts made by individual ssDNA sites (Dumont *et al*. 2024). ssHiCstuff provides the first dedicated computational framework to setup a ssHi-C experiment and for processing, analyzing, and visualizing the resulting ssHi-C data from raw reads to normalized, interpretable contact profiles.

In practice, ssHiCstuff was successfully applied to:

- Designing annealing and capture oligonucleotides used to restore ssDNA on each side of a site-specific DNA double-strand break site in *S. cerevisiae.*
- Isolate and quantify probe-level contacts made by resected ssDNA regions.
- Compute normalized enrichment scores accounting for both capture efficiency and global coverage.
- Aggregate signals around functional landmarks such as centromeres and telomeres.
- Generate high-resolution 4C-like profiles.

While future development will aim to simplify certain file formats and enable standardized workflows (*e.g.*, via Nextflow), the current implementation already provides a versatile and user-friendly tool for ssHi-C experimental design and data analysis, which should facilitate adoption of the methodology by the broader research communities studying DNA metabolism in the context of 3D genome organization.
